# Complete Genome Sequence of Paraclostridium bifermentans DSM 14991

**DOI:** 10.1128/mra.00103-22

**Published:** 2022-06-27

**Authors:** G. T. Little, L. Sellés Vidal, M. Steadman, R. Leyden-Preece, G. M. Taylor, J. T. Heap

**Affiliations:** a School of Life Sciences, The University of Nottingham, University Park, Nottingham, United Kingdom; b Department of Life Sciences, Imperial College London, London, United Kingdom; c Imperial College Centre for Synthetic Biology, Imperial College London, London, United Kingdom; University of Maryland School of Medicine

## Abstract

The complete genome sequence of Paraclostridium bifermentans was obtained by assembly of Illumina and Oxford Nanopore (ONT) reads. The sequence will enable study into the organism’s ability to biohydrogenate unsaturated acyl chains in the transformation of C20 polyunsaturated fatty acids (PUFAs) into the corresponding bioactive non-methylene-interrupted fatty acids (NMIFAs).

## ANNOUNCEMENT

Paraclostridium bifermentans is a rod shaped, endospore forming, Gram-positive, obligately anaerobic, non-pathogenic bacterium (1, 2). Isolates of Paraclostridium bifermentans from areas where anophelines are endemic encode PMP1, a clostridial-like neurotoxin that selectively targets anopheline mosquitoes (3). The organism is capable of the unusual biohydrogenation of unsaturated acyl chains, transforming C20 polyunsaturated fatty acids (PUFAs) into the corresponding non-methylene-interrupted fatty acids (NMIFAs) (4), which may have novel bioactive properties (5).

Paraclostridium bifermentans type strain DSM 14991 (ATCC 638; JCM 1386; https://www.dsmz.de/collection/catalogue/details/culture/DSM-14991) was purchased from DSMZ. Spores were plated anaerobically at 37°C onto a Gifu anaerobic medium (GAM) agar plate. A single colony was grown anaerobically overnight in 10 mL GAM media, and genomic DNA was obtained by phenol-chloroform-isoamyl alcohol extraction (6). All library preparation and sequencing steps were performed by MicrobesNG (Birmingham, UK) from the same genomic DNA sample. An Illumina library was prepared using the Nextera XT library prep kit (Illumina, San Diego, USA) following the manufacturer’s protocol with the following modifications: input DNA increased 2-fold and PCR elongation time increased to 45 s. Pooled libraries were quantified using the Kapa Biosystems library quantification kit for Illumina and sequenced using a HiSeq 2500 instrument with a 250-bp paired-end protocol. Reads were adapter trimmed using Trimmomatic 0.30 (7) with a sliding window quality cutoff of Q15, to give 847,664 paired Illumina reads. Quality control (QC) reports for the Illumina data were generated using FastQC 0.11.9 (8) and aggregated using MultiQC 1.10.1 (9). Default parameters were used for all software unless otherwise specified.

A long read genomic DNA library was prepared with the Oxford Nanopore SQK-RBK004 kit using 400 to 500 ng of high-molecular-weight DNA. Barcoded samples were pooled into a single sequencing library and loaded in a FLO-MIN106 (R.9.4.1) flow cell in a GridION system (Oxford Nanopore [ONT], United Kingdom). Guppy 3.0.6 (10) was using for base calling. The ONT data were subject to QC using NanoPlot 1.38.0 (11), with mean read length, 3,770 bp; median read length, 2,384 bp; number of reads, 22,812; and read length *N*_50_, 6,390 bp.

The genome sequence was assembled using Unicycler 0.4.8 (12) with parameters min_fasta_length 2000 and bold mode, with the chromosome split across 3 incomplete contigs due to incomplete long read coverage. To achieve a single circular contig, two 40 kbp sequences from Clostridium bifermentans strain cbm (accession number NZ_CP032452.1; 2067654..2107654, 2123574..2163574) were added as long read scaffolds. All scaffold sequences were updated during the assembly by short-read Illumina data from DSM 14991. The assembly produced 5 complete circular contigs (Table 1). The assembly was subject to QC by visualization of the assembly graphs with Bandage 0.8.1 (13). Annotation was done using the NCBI Prokaryotic Genome Annotation Pipeline (PGAP) 5.2 (14).

**TABLE 1:** Length and NCBI accession numbers of the sequenced chromosome and plasmids

Name	Length (bp)	NCBI accession
Chromosome	3,304,779	CP079737.1
Plasmid unnamed1	142,041	CP079738.1
Plasmid unnamed2	52,569	CP079739.1
Plasmid unnamed3	47,028	CP079740.1
Plasmid unnamed4	19,800	CP079741.1
Total	3,566,217^*a*^	

a GC content of 28.6%.

### Data availability.

The nucleotide sequences of the Paraclostridium bifermentans DSM 14991 chromosome and plasmids have been deposited in NCBI GenBank under accession numbers CP079737 to CP079741. Sequencing reads have been deposited in the NCBI Sequence Read Archive (SRA) under accession numbers SRX11627627 and SRX11627626.
